# Does Tranexamic Acid Administration Increase the Risk of Thromboembolism?

**DOI:** 10.7759/cureus.69334

**Published:** 2024-09-13

**Authors:** Ibrahim Al Babtain, Khalid H Alhadlaq, Ziad A Aljaafri, Abdullah Alhaqbani, Abrar Al-Mutairi, Heythem AlZamel, Khaled Albedah, Raed Alshalfan, Rifan Alyami, Sami Almalki

**Affiliations:** 1 General Surgery, Ministry of National Guard Health Affairs (MNGHA), Riyadh, SAU; 2 General Surgery, King Abdullah International Medical Research Center, Riyadh, SAU; 3 College of Medicine, King Saud Bin Abdulaziz University for Health Sciences, Riyadh, SAU; 4 Research, College of Applied Medical Sciences, King Saud Bin Abdulaziz University for Health Sciences, Riyadh, SAU; 5 Research, King Abdullah International Medical Research Center, Riyadh, SAU

**Keywords:** hemorrhage, saudi arabia, tranexamic acid (txa), trauma, venous thromboembolism (vte)

## Abstract

Introduction

Hemorrhage following trauma is one of the leading causes of death worldwide. Venous thromboembolism (VTE) is a common complication of an increased coagulable state, and the risk of VTE following the administration of tranexamic acid (TXA), an antifibrinolytic agent, is still controversial. Our aim is to understand whether there is any association between the administration of TXA and the risk of VTE development.

Methods

A retrospective cohort study enrolled trauma patients presenting to a level 1 trauma center who received TXA at King Abdulaziz Medical City (KAMC), a tertiary hospital in Riyadh, Saudi Arabia, between 2016 and 2020. Variables included were patients’ demographics, comorbidities, laboratory investigations, TXA’s route of administration, date of administration, and dose. Finally, the development of VTE in the form of deep vein thrombosis (DVT) or PE and those who died were evaluated.

Results

A total of 361 adult trauma patients who received TXA were included in the study. Most were males (90.3%) with an average age of 33 years and had a normal BMI (43.3%). Nine patients (2.5%) developed DVT, one of whom died, and five patients (1.4%) developed PE, with no recorded deaths.

Conclusion

The risk of VTE following the administration of TXA remains controversial and yet to be clearly demonstrated. In this study, there was no significant association between TXA administration and the risk of developing VTE. More research with a larger sample size is needed to identify and recognize any significant risk factors for developing such a condition.

## Introduction

Trauma is still one of the leading causes of death worldwide, and hemorrhage is the leading cause of death following trauma, accounting for over 80% of operating room deaths and 50% of deaths in the first 24 hours after reaching a trauma center [1-2]. Tranexamic acid (TXA) is an antifibrinolytic agent that plays a major role in controlling hemorrhages in trauma patients. It acts as a competitive inhibitor of fibrinolysis [3-4]. In some studies, TXA administration was associated with lower rates of mortality following trauma [3-4]. On the other hand, other studies have demonstrated no significant effect of TXA on mortality for trauma patients [5-6]. Due to TXA’s mechanism of action, it may be associated with an increased risk of developing venous thromboembolism (VTE) [7-8]. However, in recent studies, there was an increased percentage of VTE in patients who received TXA, but no statistically significant association; this study was not done on trauma patients [9]. In the HALT-IT study, there was an association between TXA administration and VTE; however, this study was conducted on patients with acute GI bleeding [10]. As is known, VTE is a common complication of an elevated coagulable state. It typically occurs when a clot forms in the deep veins and presents in conditions such as deep venous thrombosis (DVT) and pulmonary embolism (PE) [11].

The risk of VTE following the administration of TXA is still not well understood and has yet to be demonstrated clearly. However, previous research has emphasized the importance of the early use of TXA and how efficient it is in reducing mortality rates, despite any other risks [12]. In a recent international multicenter study, no link was found between the administration of TXA and VTE; on the contrary, the use of TXA was associated with better outcomes and lower rates of blood transfusion [13]. Similarly, a systematic review conducted by Taeuber et al. showed no relationship between TXA administration and VTE [14]. On the other hand, a recent cohort study that examined the use of TXA as an independent risk factor for VTE concluded that it might be a risk factor; however, it was not associated with any survival benefit [7].

There is limited research investigating the utilization of TXA and its effects on VTE. To tackle these issues, the current study was conducted to investigate the use of TXA and whether it is an independent risk factor for developing VTE in trauma patients at the National Guard Hospital (NGH) in Saudi Arabia.

## Materials and methods

A retrospective cohort study was conducted at King Abdulaziz Medical City (KAMC), a tertiary hospital in Riyadh, Saudi Arabia. The sample size was determined by including all trauma patients who received TXA from January 2016 to December 2020. Patients who did not meet the inclusion criteria were excluded. The study was approved by the Institutional Review Board (IRB) at King Abdullah International Medical Research Centre (NRC21R/507/11).

Data were collected through the medical health records system called BESTCare at KAMC. We used a data collection sheet that was prepared by the research team based on the data of interest. The data collection sheet included demographics, comorbidities, laboratory results, nature of injury, date of TXA administration (in the hospital, not prehospital), route of TXA administration, and dose of TXA, all of which were gathered at the time of presentation in the emergency department. The standard dose of TXA in a trauma setting, as reported in the literature, is 1 g followed by another gram if needed, 8 hours apart [15]. Finally, the incidence of VTE (DVT or PE) was measured. The diagnosis of PE was confirmed through chest computed tomography angiography (CTA) and DVT through lower limbs Doppler USG. Other forms of hypercoagulable states such as myocardial infarctions (MI), strokes, portal vein thrombosis, or pulmonary thrombosis were not calculated in our study.

All data were collected through Microsoft Excel (Microsoft Corp., Redmond, WA, USA) and transferred for analysis. The data were checked for any missing information, and new variables were recorded and computed based on the extracted data.

Statistical analysis was performed using IBM SPSS Statistics version 25.0 (IBM Corp., Armonk, NY, USA). Frequencies and percentages were used to detail categorical variables, whereas continuous variables were examined with mean and standard deviation. Fisher’s exact test was conducted to determine the associations between patients who developed VTE (including DVT and PE) and certain predictors (age, gender, BMI, comorbidities, laboratory results, dose, and death). A p-value <0.05 was considered statistically significant.

## Results

A total of 361 adult patients who were admitted to the trauma center at NGH and received TXA were included in this study, most of whom were males (90.3%), with an average age of 33 years (ranging from 14 to 79). Most of the patients had a BMI within the normal range (43.4%), while 38.8% were classified as overweight. Diabetes was the most common comorbidity (65.1%), followed by hypertension (31%) (Table 1).

**Table 1:** Baseline characteristics of treated patients with TXA. TXA: Tranexamic acid; CKD: Chronic Kidney Disease; PVD: Peripheral Vascular Disease; HTN: Hypertension; DM: Diabetes Mellitus.

Demographic data	(N%)
Age	
<18	19 (5.3%)
18-30	193 (53.5%)
30-45	89 (24.7%)
45-60	34 (9.4%)
>60	26 (7.1%)
Gender	
Male	326 (90.3%)
Female	35 (9.7%)
BMI	
Underweight	14 (3.9%)
Normal weight	152 (43.4%)
Overweight	140 (38.8%)
Obesity	50 (13.9%)
Comorbidities	
DM	
Yes	235 (65.1%)
No	126 (34.9%)
HTN	
Yes	112 (31.0%)
No	249 (69.0%)
Hypothyroidism	
Yes	2 (0.6%)
No	359 (99.4%)
Anemia	
Yes	1 (0.3%)
No	360 (99.7%)
PVD	
Yes	1 (0.3%)
No	360 (99.7%)
CKD	
Yes	6 (1.9%)
No	355 (98.1%)

In this study, the incidence of VTE was divided into DVT and PE. Nine patients (2.5%) developed DVT, the majority of whom were males (77.8%). The only significant comorbidity detected in these patients was hypothyroidism, with a p-value of 0.04. Among these patients, one death (11.1%) was recorded. The associations between demographic variables, laboratory findings, and outcomes of DVT are presented in Table 2.

**Table 2:** Demographic variables, laboratory findings, and outcomes with DVT. DVT: Deep Vein Thrombosis; DM: Diabetes Mellitus; HTN: Hypertension; PVD: Peripheral Vascular Disease; CKD: Chronic Kidney Disease; INR: International Normalized Ratio; PT: Prothrombin Time; PTT: Partial Thromboplastin Time; HgB: Hemoglobin.

DVT	DVT		P-value
	Yes N (%)	No N (%)	
Age			
<18	0 (0.0%)	19 (5.4%)	0.39
18-30	5 (55.6%)	188 (53.4%)	
30-45	1 (11.1%)	88 (25.0%)	
45-60	1 (11.1%)	33 (9.4%)	
>60	2 (22.2%)	24 (6.8%)	
Gender			
Male	7 (77.8%)	319 (90.6%)	0.21
Female	2 (22.2%)	33 (9.4%)	
BMI			
Underweight	0 (0.0%)	14 (4.0%)	0.51
Normal weight	4 (44.4%)	148 (42.7%)	
Overweight	5 (55.6%)	135 (38.9%)	
Obesity	0 (0.0%)	50 (14.4%)	
Comorbidities			
DM			
Yes	3 (33.3%)	229 (65.1%)	0.61
No	6 (66.7%)	123 (34.9%)	
HTN			
Yes	1 (11.1%)	111 (31.5%)	0.28
No	8 (88.9%)	241 (68.5%)	
Hypothyroidism			
Yes	1 (11.1%)	1 (0.3%)	0.04
No	8 (88.9%)	351 (99.7%)	
Anemia			
Yes	0 (0.0%)	1 (0.3%)	0.97
No	9 (100%)	351 (99.7%)	
PVD			
Yes	0 (0.0%)	1 (0.3%)	0.97
No	9 (100%)	351 (99.7%)	
CKD			
Yes	0 (0.0%)	6 (1.7%)	0.85
No	9 (100%)	346 (98.3%)	
Lab result			
INR: Mean (SD), Units	1.18 ± 1.12	1.18 ± 0.25	0.92
PT: Mean (SD), Units	12.56 ± 1.21	12.7 ± 2.48	0.83
PTT: Mean (SD), Units	30.76 ± 7.75	29.02 ± 12.70	0.68
HgB: Mean (SD), Units	122.11 ± 26.89	134.80 ± 26.57	0.15
Platelet: Mean (SD), Units	238.66 ± 178.14	278.57 ± 86.97	0.52
Dose			
<1g	9 (2.6%)	0 (0.0%)	0.97
≥1g	343 (97.4%)	9 (100%)	
Mortality			
Non-survivor	1 (11.1%)	68 (19.3%)	0.46
Survivor	8 (88.9%)	284 (80.7%)	

Out of 361 trauma patients, only five patients (1.4%) developed PE. All were male, and no deaths were recorded. There were no significant associations between the established predictors and the development of PE (Table 3).

**Table 3:** Demographic variables, laboratory findings, and outcomes with PE. PE: Pulmonary Embolism; DM: Diabetes Mellitus; HTN: Hypertension; PVD: Peripheral Vascular Disease; CKD: Chronic Kidney Disease; INR: International Normalized Ratio; PT: Prothrombin Time; PTT: Partial Thromboplastin Time; HgB: Hemoglobin.

PE	PE		P-value
	Yes N (%)	No N (%)	
Age			
<18	1 (20.0%)	18 (5.1%)	0.55
18-30	3 (60.0%)	190 (53.4%)	
30-45	1 (20.0%)	88 (24.7%)	
45-60	0 (0.0%)	34 (9.6%)	
>60	0 (0.0%)	26 (7.2%)	
Gender			
Male	5 (100%)	321 (90.2%)	0.59
Female	0 (0.0%)	35 (9.8%)	
BMI			
Underweight	0 (0.0%)	14 (4.0%)	0.38
Normal weight	4 (80.0%)	148 (42.2%)	
Overweight	1 (20.0%)	139 (39.6%)	
Obesity	0 (0.0%)	50 (14.2%)	
Comorbidities			
DM			
Yes	4 (80.0%)	231 (64.9%)	0.42
No	1 (20.0%)	125 (35.1%)	
HTN			
Yes	2 (40.0%)	110 (30.9%)	0.49
No	3 (60.0%)	246 (69.1%)	
Hypothyroidism			
Yes	0 (0.0%)	2 (0.6%)	0.97
No	5 (100%)	354 (99.4%)	
Anemia			
Yes	0 (0.0%)	1 (0.3%)	0.98
No	5(100%)	355 (99.7%)	
PVD			
Yes	0 (0.0%)	1 (0.3%)	0.98
No	5 (100%)	355 (99.7%)	
CKD			
Yes	0 (0.0%)	6 (1.7%)	0.91
No	5 (100%)	350 (98.3%)	
Lab result			
INR: Mean (SD), Units	1.39 ± 0.49	1.18 ± 0.24	0.92
PT: Mean (SD), Units	15.02 ± 5.21	12.70 ±2.39	0.83
PTT: Mean (SD), Units	23.60 ± 14.37	29.14 ±12.57	0.68
HgB: Mean (SD), Units	116.00 ± 28.92	134.74 ± 26.53	0.15
Platelet: Mean (SD), Units	255.40 ± 84.51	277.88 ± 90.28	0.52
Dose			
<1g	0 (0.0%)	9 (2.5%)	0.31
≥1g	5 (100%)	347 (97.5%)	
Mortality			
Non-survivor	0 (0.0%)	69 (19.4%)	0.34
Survivor	5 (100%)	287 (80.6%)	

## Discussion

Trauma-related hemorrhage is one of the leading causes of death in trauma centers worldwide. Evidence from large-scale randomized trials has demonstrated that TXA is one of the most crucial fibrinolytic agents used in the trauma setting to stop bleeding. The aim of this study was to investigate whether there is any association between administering TXA, an antifibrinolytic agent that plays a role in controlling hemorrhage in trauma patients, and experiencing a thromboembolic event, such as DVT or PE.

In this study, 90.3% of the patients were male, which could be attributed to the relatively higher prevalence of trauma among males in our region, as already reported in a recently conducted study that showed the prevalence of trauma among males to be more than 75% [16-17].

Regarding age distribution, almost 60% were under the age of 30, 25% of patients were aged 30 to 45 years, and approximately 15% were above 45 years of age. Similar results were found in another study conducted in Korea, which showed a comparable age group distribution, with an average age of 35 years [17].

Different coexisting conditions may contribute to the risk of developing further complications after traumatic injuries. In this study, the comorbid conditions that were reported at the time of injury were studied and analyzed. Two-thirds of the patients in this study were reported to have diabetes, and one-third were found to have hypertension. To a lesser extent, the least reported comorbidities were anemia and peripheral vascular disease, at 0.3% each, followed by hypothyroidism in 0.6% of patients, and finally, approximately 2% of patients were found to have chronic kidney disease. In support of the findings of this study, among several other studies that analyzed comorbidities in elderly age groups, one study showed that trauma patients over the age of 55 had a higher incidence of hypertension and diabetes [18].

Only nine (2.5%) patients developed a DVT, with the majority being males (77.8%). Almost 55% of those with DVT were under the age of 30. The only significant comorbidity detected was hypothyroidism (p-value = 0.04). The remaining comorbidities did not show any significance for the development of DVT. Regarding laboratory studies, including International Normalized Ratio (INR), prothrombin time (PT), partial thromboplastin time (PTT), hemoglobin level, and platelet count, no significant associations were established. Of the nine patients who had DVT, one death was recorded. Regarding the dosage of TXA administered to the patients who developed DVT, all were given more than one gram at the time of injury. Contrary to a recent multicentric prospective study, our study did not show any association between TXA administration and VTE, which could be because of our smaller sample size, not including other forms of VTE like pulmonary thrombosis, portal vein thrombosis, strokes, or MI [19].

Five (1.4%) patients developed PE, but no significant comorbidities were reported. INR, PT, PTT, hemoglobin level, and platelet count were among the laboratory tests requested for these patients, and they did not reveal any relevance. No deaths were recorded for the PE patients. Regarding the dosage of TXA administered to these patients, all received more than one gram at the time of injury. Internationally, some previous research utilized different methods of evaluating the association between TXA administration and the risk of VTE in its combined form rather than analyzing DVT and PE separately. One study showed that the risk of developing a VTE was higher in those who received TXA: 11.4% of patients who received TXA developed a VTE, whereas only 7.4% of those who did not receive TXA developed a VTE [7].

The type of injury can play a crucial role in the development of VTE. Patients at risk of bleeding will receive TXA as standard, either prehospital or upon admission; however, patients who have tendencies of bleeding even after admission will not receive prophylactic anticoagulant. An example of this is patients who have severe traumatic brain injury; even after stabilization, anticoagulants will be contraindicated for a period of time due to the risk of rebleeding. Some of these patients will not even receive mechanical prophylaxis, as some of them will not be able to ambulate or have lower limbs fractures, preventing the possibility for lower limbs pneumatic compression application [20].

There are some limitations to the current study. The fact that information was obtained from only one center is one of the article's drawbacks because it might limit how broadly the insights can be applied. To determine whether the administration of TXA may be linked to an elevated risk of developing a VTE, this topic needs further investigation with a larger sample size and the involvement of several trauma centers, including more information about the time of administration, type of injury, and other forms of VTE.

## Conclusions

VTE is a common complication of a heightened coagulable state. The risk of VTE following the administration of TXA is still controversial and has yet to be demonstrated clearly, even though multiple studies have been conducted on this topic. However, many studies have emphasized the importance of early use of TXA and its efficiency in reducing mortality rates, despite any other risks. In our study, there was no significant association between TXA administration and the risk of developing a VTE. An increased number of trauma patients led to the frequent use of TXA without clear evidence of VTE development. Therefore, more research is needed to identify and recognize any significant risk factors for developing such a condition.
